# Evaluating the Impact of N-Glycan Sequon Removal in the p27 Peptide on RSV F Protein Immunogenicity and Functionality

**DOI:** 10.3390/v16121848

**Published:** 2024-11-28

**Authors:** Lotte Jacobs, Annelies Leemans, Kim Stobbelaar, Axelle Fransen, Paul Cos, Peter Delputte

**Affiliations:** 1 Laboratory for Microbiology, Parasitology and Hygiene, Infla-Med Centre of Excellence, University of Antwerp (UA), Universiteitsplein 1 S.7, 2610 Antwerp, Belgium; lotte.jacobs@uantwerpen.be (L.J.); kim.stobbelaar@uantwerpen.be (K.S.); axelle.fransen@uantwerpen.be (A.F.); paul.cos@uantwerpen.be (P.C.); 2 Pediatrics Department, Antwerp University Hospital (UZA), Wilrijkstraat 10, 2650 Edegem, Belgium

**Keywords:** RSV, fusion protein, p27 peptide, N-glycosylation, DNA immunization

## Abstract

Respiratory syncytial virus (RSV) is the leading cause of acute lower respiratory tract infections in young children, elderly and immunocompromised patients worldwide. The RSV fusion (F) protein, which has 5–6 N-glycosylation sites depending on the strain, is a major target for vaccine development. Two to three of these sites are located in the p27 peptide, which is considered absent in virions. Prior research from our group showed that removing the N-glycan at position 116 (N116) in p27 led to higher neutralizing antibody responses and better protection against RSV. In this study, the effect of single, double and triple N-glycan deletion mutations in F p27 was evaluated. Surprisingly, all mutants exhibited similar expressions and functionality to the wild-type F protein. All F p27 glycomutants induced neutralizing antibodies and lowered lung viral loads after an RSV challenge in a mouse model. Although N-glycans in p27 influence immune responses, their exact role in RSV biology remains unclear. Possibly, these glycans, which are mostly conserved, play a role in other aspects of virus replication and biology.

## 1. Introduction

Respiratory syncytial virus (RSV) is the leading cause of acute lower respiratory tract infections (ALRIs) worldwide, including bronchiolitis and pneumonia, among children below the age of five. In immunocompromised adults and the elderly, RSV infections also form a significant problem, with RSV being the second most important viral cause of significant respiratory disease in adulthood [1,2]. It is estimated that there are 33 million cases with 3.4 million hospitalizations and up to 150,000 deaths globally each year [3]. Current treatment options remain restricted to supportive care, despite the high medical and societal burden. Palivizumab (Synagis^®,^AstraZeneca, Cambridge, United Kingdom), a humanized monoclonal antibody (mAb) for passive immunization of high-risk infants which targets the fusion (F) protein, has been the only FDA-approved immunoprophylaxis for a long time [4]. Recently, nirsevimab (Beyfortus^TM^, Sanofi, Paris, France and AstraZeneca, Cambridge, United Kingdom), a new long-acting mAb which can be used in all children up to 12 months old for prevention of RSV during their first season and high-risk infants during their second season, has been registered for use in the European Union and the UK and will be available in a single-shot regimen as of next winter (2024–2025) [5]. The vaccine landscape has also undergone significant changes in the last few years. A growing number of RSV vaccine candidates are at different stages of development targeting different populations (elderly, pregnant women and children). This has resulted in FDA approval for three vaccines, namely Pfizer’s Abrysvo, GSK’s Arexvy and Moderna’s mResvia [5,6,7]. All of these vaccines are based on the stabilized prefusion F protein. However, developing vaccines for children remains a significant challenge.

RSV is a non-segmented, negative sense, single-stranded RNA virus belonging to the genus *Orthopneumovirus* in the family Pneumoviridae [8]. Its genome is approximately 15.2 kb large and encodes 11 proteins, 3 of which are displayed on the viral envelope: the attachment (G) protein, the F protein and the small hydrophobic (SH) protein [2,9]. The RSV F protein is responsible for fusion of the viral envelope with the cellular membrane and is essential for viral entry, spread and replication [9,10]. It is a major target for neutralizing antibodies [11,12] and is the most conserved RSV envelope protein with amino acid (aa) sequence identities of 90% or higher [9].

The RSV F protein is a type I glycoprotein which is initially transcribed as an inactive precursor (F0) and post-translationally modified by furin cleavage into disulfide-linked subunits F1 (50 kDa) and F2 (20 kDa) with removal of a 27 aa peptide (p27), resulting in the mature and biologically active F protein [13,14]. This active F protein exists in the prefusion conformation, which readily rearranges in the post-fusion conformation upon fusion. RSV F is co- and post-translationally modified by the attachment of N-linked glycans to asparagine (N) residues within a consensus sequence (N-X-S/T) [15,16,17]. Depending on the strain, five or six potential N-glycosylation sites are present, of which two are located in the F2 subunit (N27 and N70), two or three additional sites are located in the intervening peptide p27 (N116, N120 and N126), and only one site is present in the F1 subunit (N500) [18].

N-glycosylation of glycoproteins is an important post-translational modification which is involved in several processes determining their structure and activity and plays a role in proper folding and subsequent trafficking of the protein [19,20]. Furthermore, N-glycans can influence translocation to the cell surface and interaction with host cells [19]. For example, for RSV, it was demonstrated that the N-glycan positioned at N500 at the RSV F protein is important for fusion activity, and the syncytial size and frequency significantly reduce upon removal of this N-glycan [21,22,23]. In addition, N-glycans can influence the immunogenicity of viral proteins by shielding antigenic sites on the proteins, thereby affecting the recognition of antibodies, as was demonstrated for human influenza A virus hemagglutinin [24]. Furthermore, immunization of mice with specific bovine RSV glycomutants elicited higher neutralizing antibody titers compared with the wild-type (WT) F protein [25].

Previous work from our group demonstrated enhanced antibody responses when BALB/c mice were immunized with human RSV F DNA glycomutant constructs, especially after an RSV challenge. In these constructs, the N-glycans at positions N27, N70, N116, N126 and N500 were deleted by replacing asparagine (N) codons (AAT/AAC) at the N-glycosylation sites with glutamine (Q) codons (CAA/CAG), thus changing the consensus sequence N-X-S/T into Q-X-S/T, resulting in single (N27Q, N70Q, N116Q, N126Q and N500Q), triple (N27-70-500Q) and quintuple (N27-70-116-126-500Q) glycomutants. Surprisingly, the highest antibody responses were elicited in the mice immunized with the F DNA construct that lacks the N-glycan sequon for N116, which is present in the p27 peptide and therefore was assumed to be absent from the mature F protein [22].

In our previous work, only conserved N-glycosylation sites N116 and N126 in p27 were studied. However, an extra glycosylation site (N120) can be present in the p27 peptide, depending on the strain [18]. In this study, a comprehensive analysis is performed by investigating the antibody responses elicited by glycomutants within the p27 peptide, including single (N116Q, N120Q and N126Q), double (N116-120Q, N116-126Q and N120-126Q) and triple (N116-120-126Q) F p27 glycomutants. This comprises all possible combinations, and these are used to evaluate the effect of the loss of these N-glycans on cell surface expression, fusogenicity and immunogenicity, with the latter being studied through intramuscular immunization of BALB/c mice with plasmids encoding the F p27 glycomutants. A subsequent challenge with RSV A2-line19F allowed evaluation of the potential priming via DNA immunization and the protective effect of the elicited immune responses. Finally, the cross-neutralization capacities of these elicited immune responses were evaluated.

## 2. Materials and Methods

### 2.1. Cells, Virus and Antibodies

The BSR T7/5 cells were a kind gift from K.K Conzelmann (Max-von-Pettenhofer-Institut, Munich, Germany) and were grown in Glasgow’s minimal essential medium (GMEM) supplemented with 10% heath-inactivated fetal bovine serum (iFBS) and 2% minimal essential amino acids (Thermo Fisher Scientific, Waltham, MA, USA).

RSV reference strains A2 and B1 and clinical RSV isolates A2000/3-4 and A1998/3-2 were obtained from the Biodefense and Emerging Infections Research Resources Repository (BEI resources, Manassas, VA, USA), propagated in a human epithelial cell line (HEp-2) obtained from ATCC and grown in Dulbecco’s modified Eagle medium (DMEM, Thermo Fisher Scientific) supplemented with 10% iFBS. RSV cDNA-containing BAC pSynkRSV-line19F was obtained from M.L. Moore (Emory University School Of Medicine, Atlanta, GA, USA) and recovered as described before to obtain strain RSV A2-K-line19F (RSV A2l19F) [26].

Palivizumab leftovers were provided by the Paediatrics Department of the Antwerp University Hospital (Professor Verhulst). Goat anti-RSV (Virostat, Westbrook, ME, USA) and RSV reference antiserum (BEI resources) were used as polyclonal antibodies. The corresponding secondary antibodies were obtained from Thermo Fisher Scientific and included Alexa Fluor (AF) 488 goat anti-human IgG, horseradish peroxidase (HRP) conjugated goat anti-mouse and goat anti-human IgG (Thermo Fisher Scientific).

### 2.2. Construction and Expression of Recombinant RSV F Proteins

The RSV F glycosylation constructs were acquired as described before [22]. Briefly, N codons (AAT/AAC) at positions N116, N120 and N126, all situated in p27, were changed into Q codons (CAA/CAG), thereby converting N-glycosylation consensus sequence N-X-S/T into Q-X-S/T. Q is considered a good substitute for N since the side chains of Q are quite similar to those of N. Seven RSV F constructs were generated (F N116Q, F N120Q, F N126Q, F N116-120Q, F N116-126Q, F N120-126Q and F N116-120-126Q), with F WT serving as a control. Synthesis of these constructs was performed by Genscript and delivered in pUC57 simple form. Excision of the DNA from the vector was performed using appropriate restriction enzymes (New England Biolabs, Ipswich, MA, USA). The excised DNA was ligated into a pCAXL mammalian expression plasmid by using T4 DNA ligase (New England Biolabs). DNA sequencing confirmed the sequences of the recombinant RSV F proteins (VIB Genetic Service Facility, University of Antwerp). In order to express the recombinant F proteins, BSR T7/5 cells were transfected with the resulting plasmid DNA using ViaFect^TM^ Transfection Reagent (Promega, Madison, WI, USA). Briefly, the BSR T7/5 cells were seeded to reach approximately 75% confluency at the time of transfection. A 3:1 ratio of transfection reagent to plasmid DNA was used, with the result being diluted in Opti-MEM (Thermo Fisher Scientific). Transfection complexes were added to the cells in GMEM 10% iFBS after an incubation period of 20 min. The cells were then incubated at 37 °C for 24 h before further processing.

### 2.3. Immunofluorescence Analysis of Surface Expression

Plasmid DNA encoding WT F or F p27 glycomutant proteins was transfected into the BSR T7/5 cells as explained earlier, and RSV F surface expression was evaluated via confocal microscopy, measuring the fluorescence intensities. Transfected cells on coverslips in 24 well plates were incubated with polyclonal anti-RSV goat serum for 1 h at 4 °C. Afterward, the cells were fixed with 4% paraformaldehyde (PF), and goat anti-human IgG conjugated with AF488 was used to visualize the surface-expressed RSV F proteins through confocal microscopy (Leica SP8, Bethesda, MD, USA).

### 2.4. Fusion Assay

Transfection of the BSR T7/5 cells was performed as described above. After 36 h of incubation, the cells were fixed with 4% PF followed by immunofluorescence staining with palivizumab and AF488-conjugated goat anti-human IgG. The nuclei were stained with DAPI. Cells were considered syncytia when containing more than 2 nuclei. Both the syncytium frequency and mean syncytium size were determined through manual counting.

### 2.5. Immunization of Mice with Plasmid DNA

A total of 54 female 7–8 week-old BALB/c mice (Janvier Laboratories, Le Genest-Saint-Isle, France) were randomly allocated to 9 individually ventilated cages with 6 animals each. The 9 different treatment groups included pCAXL RSV F WT as a control, pCAXL F N116Q, N120Q, N126Q, N116-120Q, N116-126Q, N120-126Q, N116-120-116Q and the empty pCAXL plasmid as a negative control. Food (Carfil, Oud-Turnhout, Belgium) and drinking water were available ad libitum. On day 0 and day 21, the mice were immunized intramuscularly in both quadriceps muscles with 100 µg of endotoxin-free plasmid DNA in total (dissolved in 100 µL 0.9% NaCl solution) after anesthesia with 5% isoflurane (Halocarbon^®^, Atlanta, GA, USA). On day 0, day 21, day 35 and day 56, blood was collected via retro-orbital bleeding, and serum was obtained by clotting the blood in a serum clot activator tube (Sarstedt, Nümbrecht, Germany) for 30 min at room temperature followed by centrifugation (5 min, 10,000× *g*). The animal studies were approved by the Animal Ethical Committee of the University of Antwerp (UA-ECD 2015-63; 1 October 2015).

### 2.6. RSV Challenge

The mice were intranasally challenged with 10^6^ PFU of reference strain RSV A2-K-line19diluted in 100 µL Hanks’ balanced salt solution (HBSS) on day 56. Five days post challenge (day 61), serum was collected before the mice were sacrificed via CO_2_ asphyxiation. The lungs were removed and homogenized in HBSS for determination of the RSV RNA levels. The animal studies were approved by the Animal Ethical Committee of the University of Antwerp (UA-ECD 2015-63; 1 October 2015).

### 2.7. Antibody Responses and Neutralization Assay

The total antibody titers were determined as follows. RSV-A2-K-line19F-infected HEp-2 cells (MOI = 0.5) were used as an antigen and propagated in 96 well microtiter plates (Falcon, Corning, NY, USA,). The cells were fixed with methanol followed by permeabilization with 0.5% Triton-X-100 and blocking with 1% bovine serum albumin (BSA) (Santa Cruz Technologies, Santa Cruz, CA, USA). Heat-inactivated mice serum was added to the cells in twofold dilutions starting from a 1:10 ratio and incubated for 1 h at 37 °C. Afterward, the cells were incubated with HRP-conjugated goat anti-mouse IgG, and infection was visualized with the addition of 3,3′diaminobenzidine (DAB) (Sigma-Aldrich, St. Louis, MO, USA) as a substrate for HRP. The antibody titers of the serum were determined using light microscopic analysis of serum-based staining of the RSV-infected cells and displayed as log 2 of the lowest concentration where staining of the RSV-infected cells was observed.

Plaque reduction neutralization tests (PRNTs) were performed to determine the neutralizing antibody titers as described before [22,27]. Briefly, serial twofold dilutions of heat-inactivated serum were incubated with RSV A2-K-line19F for 1 h at 37 °C prior to inoculation of the subconfluent HEp-2 cell monolayers. Binding of the virus was allowed for 2 h at 37 °C. An overlay of DMEM + 0.6% Avicel (FMC Biopolymer, Philadelphia, PA, USA) was added to the cells, followed by 72 h incubation at 37 °C. Afterward, the cells were fixed with 4% PF, permeabilized with Triton X-100 and blocked with 1% BSA. Plaques were stained with palivizumab and HRP-conjugated goat anti-human IgG and visualized with chloronapthol (Thermo Fisher Scientific). The neutralization titers were determined by the dilution resulting in a 50% PFU reduction compared with the control wells. The 50% endpoint titers were determined by manual plaque counting.

### 2.8. Cross-Neutralization of Post-Challenge Serum

PRNTs were performed on the post-challenge serum to analyze its neutralizing capacity when challenged with different RSV strains as described above. The reference strains RSV B1 and RSV-A2-K-line19F, as well as 2 clinical RSV isolates (A2000/3-4, A1998/3-2), were used to incubate the heat-inactivated serum with before inoculation of the HEp-2 cells.

### 2.9. Determination of Lung Viral Titer by qRT-PCR

Five days post challenge (day 61), the mice were euthanized via CO_2_ asphyxiation, and their lungs were excised and homogenized in HBSS for the determination of lung RSV load through qRT-PCR. Total RNA was extracted using the High Pure RNA tissue kit (Roche, Basel, Switzerland) according to the manufacturer’s instructions and converted to cDNA using random hexamer primers and the Transcriptor First Strand cDNA synthesis kit (Roche). Then, qRT-PCR was performed to determine the relative levels of genomic RSV M cDNA using primers specific to the RSV A2 M gene (5′TCACGAAGGCTCCACATACA3′ and 5′GCAGGGTCATCGTCTTTTTC3′) and a nucleotide probe (#150 Universal Probe Library, Roche) labeled with fluorescein (FAM) at the 5′-end and with a dark quencher dye near the 3′-end. The qRT-PCR data were normalized to mRPL13A mRNA levels.

### 2.10. Statistical Analysis

Data are presented as the means ± SD of the indicated independent repeats. To determine the significance between the values of the WT group and the different glycomutant groups, the data were analyzed using one-way ANOVA and post-hoc Dunnett analysis, comparing the glycomutants versus the WT in GraphPad Prism 10.

## 3. Results

### 3.1. Expression Analysis of the RSV F Glycomutants

**Figure 1:**
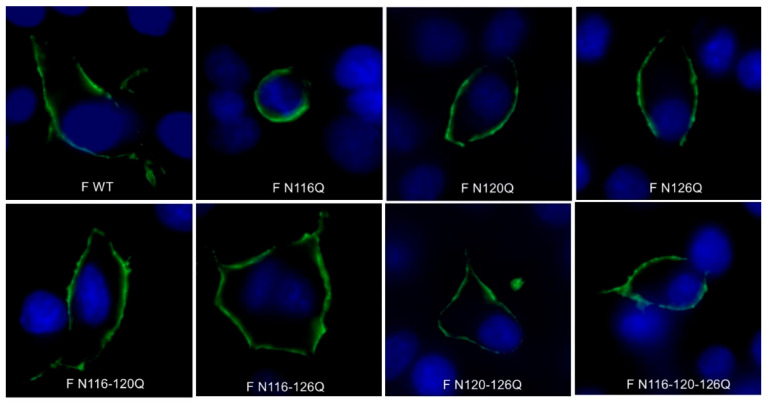
Immunofluorescence staining of cell surface expression of F p27 glycomutants. BSR T7/5 cells were transfected with pCAXL plasmid DNA encoding RSV F p27 glycosylation mutants. The nuclei were stained with DAPI (blue). F proteins are visualized with polyclonal goat anti-RSV antibodies and secondary donkey anti-goat IgG (AF488) (green). Images were acquired through confocal fluorescence microscopy (scale bar = 12 µM).

Plasmids containing the full-length F protein with single, double and triple mutations of N-glycosylation sites N116, N120 and N126 were transfected into BSR T7/5 cells. The RSV F surface expression was evaluated via confocal microscopy, and the F WT was used as a control. The results show that all F p27 glycomutants were expressed on the cell surface, suggesting no major issues with protein conformation or processing. Representative images of the immunofluorescence cell surface staining are shown in Figure 1.

### 3.2. Fusion Capacity of F p27 Glycomutants

**Figure 2:**
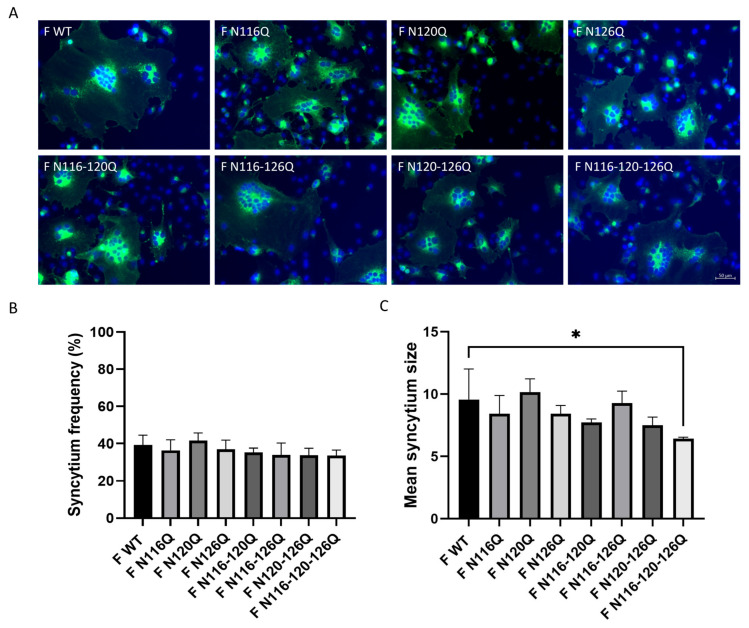
Fusion assay assessing the effect of loss of RSV F p27 N-glycans on syncytium formation. (**A**) Fluorescent images of the respective F p27 glycomutants. Nuclei were stained with DAPI (blue) and RSV F proteins were stained with palivizumab and secondary goat anti-human IgG AF488 (green) (scale bar = 50 µm). (**B**) Syncytium frequency. (**C**) Mean syncytium size. Data represent the mean ± SD of three independent repeats. * *p* < 0.05. (one-way ANOVA).

Syncytia formation was visualized through immunofluorescence staining of the RSV F protein using palivizumab (Synagis^®^) 36 h after transfection of the RSV F p27 glycomutants. The nuclei were stained with DAPI (Figure 2A). Fused cells were considered syncytia when containing more than two nuclei. All RSV F p27 glycomutants induced the formation of syncytia. Some variations were observed in the syncytium frequency between the mutants and WT, but none were significant (Figure 2B). For all RSV F p27 glycomutants, large syncytia with a mean of six nuclei or more were detectable. However, the triple mutant F N116-120-126Q-induced syncytia were significantly smaller than those of the F WT (Figure 2C).

### 3.3. Antibody Responses in Mice upon Immunization with Plasmid DNA Coding for RSV F p27-Glycomutants

**Figure 3:**
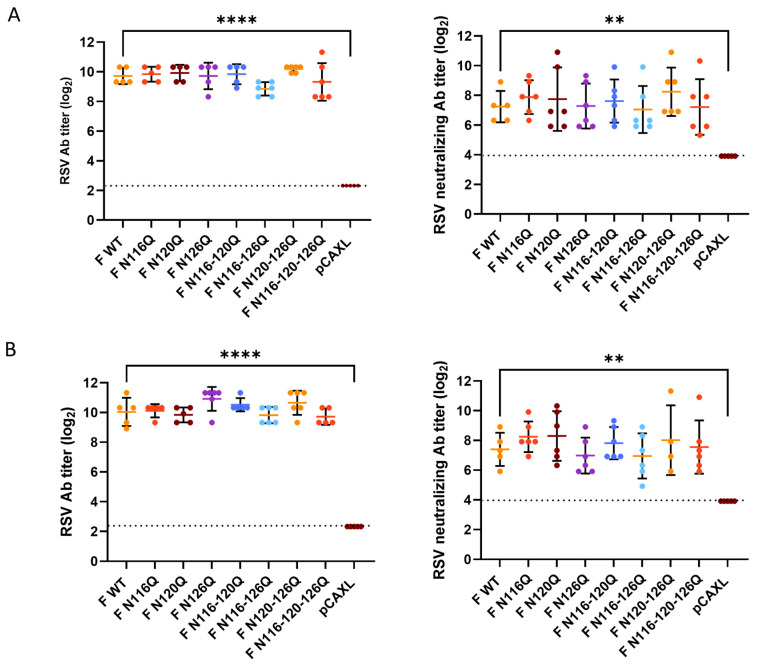
Total and neutralizing antibody responses. Two subsequent immunizations of the indicated F DNA constructs were intramuscularly administered to BALB/c mice. Serum was collected 3 weeks after prime immunization (**A**) and 2 weeks after boost immunization but before the challenge (**B**). Total (**left**) and neutralizing (**right**) antibody titers were determined. The dotted line represents the detection limit. ** *p* < 0.01. **** *p* < 0.0001 (one-way ANOVA).

First, 6–7 week-old female BALB/c mice were intramuscularly immunized with plasmids encoding glycomutant F proteins. The mice sera were collected after priming (day 0) (Figure 3A) and after boost immunization (day 21) (Figure 3B) for determination of the total and neutralizing antibody titers. In general, all F p27 constructs induced serum IgG antibodies which reacted with fixed RSV-infected HEp-2 cells.

The total serum antibody titers were determined via titration of twofold serial dilutions of heat-inactivated serum on RSV A2-K-line19F-infected HEp-2 cells. The endpoint titers were determined with light microscopic analysis. Significantly higher total antibody titers were observed for all F p27 glycomutants when compared with the empty plasmid pCAXL. However, no remarkable differences in the total antibody titers were observed between the F p27 glycomutants and F WT. Although variations in the total antibody titers were observed between different glycomutants, none of these variations were statistically significant.

Comparable trends were observed in the neutralizing antibody titers. PRNTs were performed to determine the neutralizing antibody responses. At both timepoints, significantly higher neutralizing antibody titers were observed for all F p27 glycomutants compared with the empty plasmid pCAXL. Variation in the neutralizing antibody titers elicited by different glycomutants compared with the WT was observed, but no statistical differences were found relative to the WT.

### 3.4. Neutralizing Antibody Titers and Corresponding Viral Loads After Challenge

**Figure 4:**
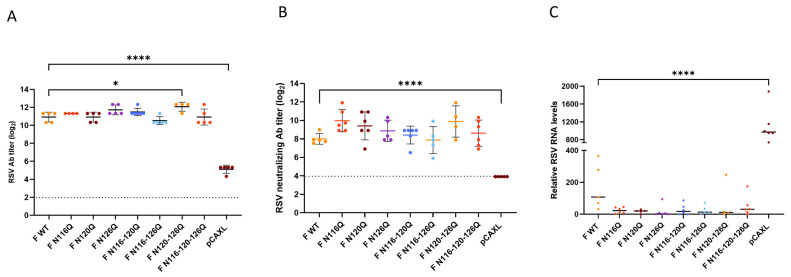
Total (**A**) and neutralizing (**B**) antibody responses and corresponding lung viral loads (**C**) after challenging BALB/c mice previously immunized with DNA. Mice were intramuscularly immunized twice with the indicated plasmids (prime immunization on day 0 and boost immunization on day 21). Five days post RSV challenge (day 61), sera were collected, and the total (**A**) and neutralizing (**B**) antibody titers were determined. Five days post challenge with 1 × 10^6^ PFU of RSV A2-K-line19F, the lungs were collected and homogenized. The relative RNA levels (**C**) in the infected lungs were determined via RT-qPCR. The dotted line represents the detection limit (**A**,**B**). * *p* < 0.05. **** *p* < 0.0001 (one-way ANOVA).

Initially, 6–7 week-old female BALB/c mice were intramuscularly immunized with plasmid DNA encoding glycomutant RSV F proteins on day 0 and day 21. Five weeks post boost immunization, the mice were intranasally challenged with RSV A2-K-line19F. The mice were sacrificed five days post challenge. The total and neutralizing antibody titers were determined as described above. Five days post challenge, significantly higher total antibody titer were observed for all F p27 glycomutants compared with the empty plasmid pCAXL (Figure 4A). The total antibody titers elicited by the F p27 glycomutants varied, with one glycomutant, F N120-126Q, showing a significantly higher titer total compared with the F WT.

Next, the neutralizing antibody titers were determined five days post challenge (Figure 4B). Significantly higher neutralizing antibody titers were observed for all F p27 glycomutants compared with the empty plasmid pCAXL. In general, all RSV F p27 glycomutants induced serum-neutralizing antibody responses at least as high as those of the F WT. However, none of the mutants induced neutralizing antibody titers which were significantly higher than those elicited by the F WT.

The lung viral loads in the lung homogenates were determined to evaluate the efficacy of F p27 glycomutant DNA immunization in providing protection and clearance of the viral infection. The relative RNA levels were determined through qRT-PCR (Figure 4C). Interestingly, all mutants showed better protection against the challenge with RSV overall, though it was non-significant.

### 3.5. Cross-Neutralization Capacity of Post-Challenge Serum

**Figure 5:**
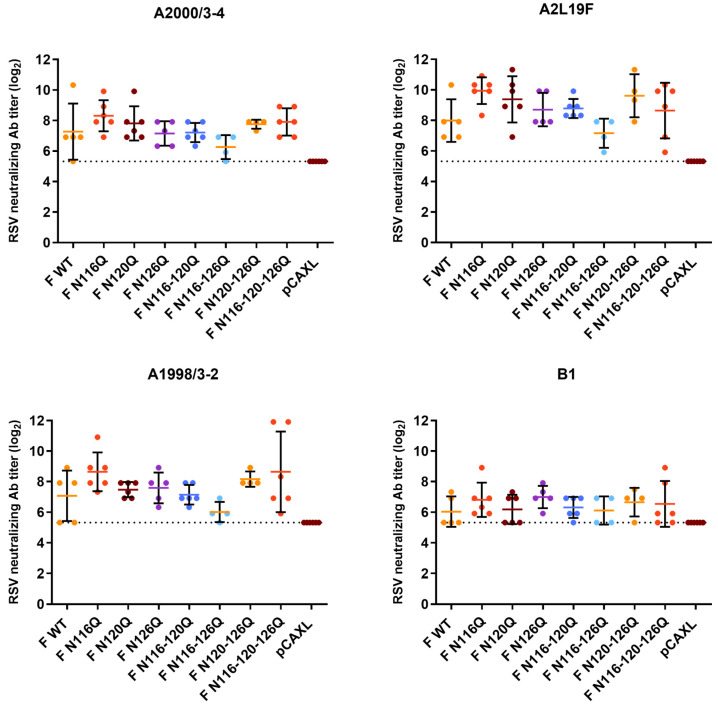
Cross-neutralization of post-challenge serum. Mice were intramuscularly immunized twice with the indicated plasmids (prime immunization on day 0 and boost immunization on day 21). Five days post RSV challenge (day 61), serum was collected, and the cross-neutralization capacities were determined with PRNTs. Dotted lines represent limit of detection (one-way ANOVA).

Sera from the 6–7 week-old female BALB/c mice who were immunized twice with F p27 glycomutant DNA (day 0 and day 21) followed by an RSV A2-K-line19F challenge (day 56) were analyzed for their cross-neutralization capacities. PRNTs were performed in which the heat-inactivated serum was incubated with two reference strains, RSV A2L19F and RSV B1, and two different clinical isolates (A2000/3-4 and A1998/3-2) before inoculation of the HEp-2 cells and subsequent determination of the neutralizing antibody titers (Figure 5).

In general, the neutralizing antibody titers tested with RSV B1 were lower than those tested with the RSV A strains. Cross-neutralization with RSV B1 resulted in no significant differences in the neutralizing antibody responses between the RSV F p27 glycomutants and F WT.

Similarly, for all RSV A strains tested (A2L19F, A2000/3-4 and A1998/3-2), no significant differences in the neutralizing antibody titers were observed for any RSV F p27 glycomutants compared with the RSV F WT.

## 4. Discussion

Although RSV was discovered in 1956, the social and medical burden remains substantial. Vaccine development has primarily focused on the RSV F protein [5,28]. F is highly conserved and a major target for neutralizing antibodies. Therefore, it is the major focus in vaccine research [11,12]. Depending on the strain, five or six N-glycosylation sites with the consensus sequence N-X-T/S are present on the RSV F protein [18,29]. At least five of these N-glycosylation sites are conserved among RSV isolates, which is suggestive of a role in the structure, function or antigenicity of the protein [15,21,30,31]. Similarly, the presence of 2–3 glycans on p27, a peptide which is assumed to be removed during maturation of the protein, is surprising.

Previous work from our group investigated the roles of five N-glycans located at positions N27, N70, N116, N126 and N500 in cell surface expression, fusogenicity, antigenicity and immunogenicity [22,31]. Surprisingly, the highest antibody responses were elicited in the mice immunized with the F DNA construct lacking the N-glycan sequon for N116, which is present in the p27 peptide and is generally assumed to be absent from the mature F protein [22].

Here, we conducted a more detailed study in which the three N-glycosylation sites in the p27 peptide of the RSV F protein of strain line 19 were abolished by N to Q codon changes, resulting in single (N116Q, N120Q and N126Q), double (N116-120Q, N116-126Q and N120-126Q) and triple (N116-120-126Q) mutants [22]. Also, higher antibody responses were observed for N116Q in this study, though they did not differ significantly from the other F p27 glycomutants or WT F.

For a long time, it was accepted that the peptide p27 is absent from the mature F protein, because of the double furin cleavage upon expression is considered essential to allow transport of the F protein to the cell surface [13]. Therefore, p27 was considered not to be exposed to the immune system and not a target for neutralizing antibodies targeting viral particles [32]. However, published data on when p27 is fully removed from F are conflicting [18]. While some considered p27 removal essential for monomers to associate into the compact trimer [33], other data suggest a partial cleavage of the mature RSV F protein in which trimer formation precedes the second cleavage and removal of the p27 peptide. In this case, the first cleavage happens during transport to the cell surface followed by a second cleavage event that occurs at a later stage, possibly during virus entry, most likely by a furin-like enzyme. This would mean that p27 can be present on the virion surface and the surface of the infected cells, thereby being exposed to immune recognition [14,34]. Indeed, an immunodominant epitope in F p27 was identified in young children and some studies show that p27 specific antibodies are present in some populations, but to what extent this plays a role in protection is not clear [35,36,37].

Variations in N-glycosylation could lead to differences in F protein processing [19,20]. Since N-glycosylation can play a role in proper protein folding, the surface expression of F p27 glycomutants was determined. All F p27 glycomutants were expressed at the cell surface after transfection of BSR T7/5 cells, suggesting that deletion of N-glycans in p27 does not critically affect protein conformation or processing. Next, the fusion activity of F p27 glycomutants was studied. In contrast to what was already demonstrated for the N500 glycomutant by Zimmer et al. and also confirmed by our group, all F p27 glycomutants induced fusion, with minor differences compared with the F WT, except for the triple F p27 glycomutant, which induced significantly smaller syncytia. All F p27 glycomutants induced syncytia of six or more nuclei, indicating that N-glycans at the respective positions are not a requirement for fusion activity [21,23]. No significant differences were seen in the fusion frequency when comparing the single, double and triple F p27 glycomutants to the F WT, suggesting that changes in the N-glycans in p27 do not affect the fusion mechanism.

Another well-known role of viral glycans is their glycan shielding ability [24,38,39,40,41]. P27 glycans could mask antigenic sites, thereby altering the antibody response. Lee et al. (2022) demonstrated that two peptides (aa 101–121 and 110–136) spanning the p27 domain controlled viral loads with a significantly reduced lung histopathology, demonstrating a potential role for p27 in protective immunity [34]. In this study, after immunization of BALB/c mice with the different F p27 glycomutants, no significant differences were observed in the total and neutralizing antibody titers. However, following a challenge with RSV A2L19F, all antibody titers increased, revealing more variability between the RSV F p27 glycomutants, though these differences remained non-significant. Together with an increase in the neutralizing antibody titers, a decrease in the viral RNA loads for all F p27 glycomutants was observed. In line with our previously published data on N116 [22], F N116Q demonstrated strong performance in both neutralizing antibody titers and reducing viral loads post challenge, though this was non-significant. A possible explanation for this observation is that removal of N-glycans exposes new neutralizing epitopes or may increase access to existing epitopes [42]. It is plausible that N-glycans in the p27 region might reduce glycan masking and therefore expose otherwise less prominent epitopes. Alternatively, glycan consensus sequences can also be part of an antigenic site [43]. Since p27 is only 27 aa large, all three positions for N-glycosylation are within a short distance from the furin cleavage sites (site I, RARR_109_, and site II, KKRKRR_136_) [13], and the presence or absence of bulky, branched N-glycans may affect the accessibility of furin enzymes, potentially affecting cleavage efficiency. This in turn could increase the exposure of strongly neutralizing epitopes at sites V and Ø [33,44]. Most likely, such effects would not be all or nothing, which may explain the variability in the obtained results. An important limitation of this study is the lack of detailed biochemical characterization of both WT F and p27 glycomutant F. Assessing the precise structural features of the N-glycans, including their structure and site occupancy, would offer valuable insights, as variations in N-glycan occupancy could explain the discrepancies observed in the results.

RSV can be divided into two subgroups, RSV A and RSV B, which are further divided into lineages [45]. RSV A and B co-circulate during the RSV season, with one subtype predominating [46,47]. In this study, we evaluated the cross-neutralization capacities of the serum collected from mice immunized with F p27 glycomutants and challenged by RSV A2-K-line19F. Three RSV A isolates and one RSV B isolate were used to evaluate the breadth of protection. Sera from all mice succeeded in neutralizing the different RSV isolates, indicating that the antibodies elicited by F p27 glycomutant immunization recognize the different RSV strains. This is of importance, considering the co-circulation of different genotypes. Remarkably, the neutralizing antibody titers were lower for RSV B. This was also observed in a recent study on human adult serum [37]. A possible explanation might lie in the different antigenicity between RSV A and RSV B. Although the main sequence differences are in the G protein, the F protein also contains significant variation in the sequence between RSV A and RSV B [48,49]. It is important to note that for the construction of F p27 glycomutants, the backbone of RSV A2 line19 was used, which might have influenced the recognition of RSV B strains by antibodies.

Although several vaccines targeting the F protein locked in the prefusion conformation have been accepted, our knowledge about the structure of the F protein is still incomplete [50]. It is not fully understood where and if p27 is completely excised or what the role of p27 is in protective immunity. It is clear that p27 is not fully removed in some conditions and that p27 may be important for immunogenicity of the F protein and possibly protective immunity [36,37]. It has also been demonstrated that p27 cleavage is dependent on the cell line and RSV subtype used, which might be explained by different expression levels of furin-like proteases, or the involvement of other prohormone convertases [51]. With regard to the N-glycans which remain conserved at p27, this study demonstrated that removal of the conserved N-glycans positioned at p27 did not result in obvious alterations in F protein folding, expression or functionality in vitro. As was suggested in our previous work [22], N-glycans located at the p27 peptide might modulate immune responses. In these in vivo experiments, subtle differences were observed between the different F p27 glycomutants and the F WT. However, even the removal of three N-glycans located within p27 did not significantly alter the immune response in the mice. Since no remarkable differences were observed in this study, it remains unclear what the role of these p27-N-glycans in RSV F biology is, but the fact that they were conserved strongly favors a critical role. Future studies should focus on exploring the biochemical properties of p27 glycomutants, as it remains uncertain whether the glycans are indeed present in WT F and p27 glycomutants, given that these are theoretical glycosylation sites. Conducting glycan profiling will be essential to enhance our understanding of the glycans which are actually present. This information could provide valuable insights into the role of these glycans in RSV F biology and their potential impact on immune responses. Possibly, these glycans may only be of importance for natural infection in humans via mechanisms which cannot be recapitulated with in vitro experiments or in mice.

## Data Availability

The dataset supporting the conclusions of this article is included within the article.
